# Short-term evaluation of tegumentary changes of the nose in oral breathers undergoing rapid maxillary expansion^☆^

**DOI:** 10.1016/j.bjorl.2017.05.010

**Published:** 2017-06-26

**Authors:** Fauze Ramez Badreddine, Reginaldo Raimundo Fujita, Mario Cappellette

**Affiliations:** Universidade Federal de São Paulo (UNIFESP), Escola Paulista de Medicina (EPM), Departamento de Otorrinolaringologia – Cirurgia de Cabeça e Pescoço, São Paulo, SP, Brazil

**Keywords:** Nose, Soft-tissue, Maxillary expansion, Multislice computed tomography, Nariz, Tecidos moles, Expansão maxilar, Tomografia computadorizada multislice

## Abstract

**Introduction:**

Rapid maxillary expansion is an orthodontic and orthopedic procedure that can change the form and function of the nose. The soft tissue of the nose and its changes can influence the esthetics and the stability of the results obtained by this procedure.

**Objective:**

The objective of this study was to assess the changes in nose dimensions after rapid maxillary expansion in oral breathers with maxillary atresia, using a reliable and reproducible methodology through computed tomography.

**Methods:**

A total of 30 mouth-breathing patients with maxillary atresia were analyzed and divided into a treatment group who underwent rapid maxillary expansion (20 patients, 10 of which were male and 10 female, with a *MA* of 8.9 years and a SD of 2.16, ranging from 6.5 to 12.5 years) and a Control Group (10 patients, 5 of which were male and 5 female, with a *MA* of 9.2 years, SD of 2.17, ranging from 6.11 to 13.7 years). In the treatment group, multislice computed tomography scans were obtained at the start of the treatment (T1) and 3 months after expansion (T2). The patients of the control group were submitted to the same exams at the same intervals of time. Four variables related to soft tissue structures of the nose were analyzed (alar base width, alar width, height of soft tissue of the nose and length of soft tissue of the nose), and the outcomes between T1 and T2 were compared using Osirix MD software.

**Results:**

In the TG, the soft tissues of the nose exhibited significant increases in all variables studied (*p* < 0.05), whereas, changes did not occur in the control group (*p* > 0.05). In the treatment group, mean alar base width increased by 4.87% (*p* = 0.004), mean alar width increased by 4.04% (*p* = 0.004), mean height of the soft tissues of the nose increased by 4.84% (*p* = 0.003) and mean length of the soft tissues of the nose increased by 4.29% (*p* = 0.012).

**Conclusion:**

In short-term, rapid maxillary expansion provided a statistically significant increase in the dimensions of the soft tissues of the nose.

## Resumo

**Introdução:**

A expansão rápida da maxila é um procedimento ortodôntico e ortopédico que pode alterar a forma e a função do nariz. Os tecidos moles do nariz e suas alterações podem influenciar na estética e na estabilidade dos resultados obtidos por esse procedimento.

**Objetivo:**

O objetivo desse estudo foi avaliar as alterações nas dimensões do nariz após expansão rápida da maxila em respiradores orais com atresia maxilar, usando uma metodologia confiável e reprodutível com o auxílio de tomografia computadorizada.

**Método:**

Um total de 30 pacientes respiradores orais com atresia maxilar foram avaliados e divididos em um grupo de tratamento, submetidos à expansão rápida da maxila (20 pacientes, 10 dos quais do sexo masculino e 10 do sexo feminino, com média de idade de 8,9 anos e DP de 2,16, variando de 6,5 a 12,5 anos) e um grupo controle (10 pacientes, sendo 5 do sexo masculino e 5 do sexo feminino, com média de idade de 9,2 anos, DP de 2,17, variando de 6,11 a 13,7 anos). No grupo tratado, foram realizados exames de tomografia computadorizada multislice no início do tratamento (T1) e 3 meses após a expansão (T2). Os pacientes do grupo controle foram submetidos aos mesmos exames nos mesmos intervalos de tempo. Foram analisadas quatro variáveis relacionadas às estruturas dos tecidos moles do nariz (largura da base alar, largura alar, altura do tecido mole do nariz e comprimento do tecido mole do nariz) e os resultados entre T1 e T2 foram comparados, utilizando-se o software Osirix MD.

**Resultados:**

No grupo tratado (GT), os tecidos moles do nariz apresentaram aumentos significativos em todas as variáveis estudadas (*p* < 0,05), enquanto isso não ocorreu no GC (*p* > 0,05). No GT, a largura média da base alar aumentou 4,87% (*p* = 0,004), a largura média alar aumentou 4,04% (*p* = 0,004), a altura média dos tecidos moles do nariz aumentou 4,84% (*p* = 0,003) e o comprimento médio dos tecidos moles do nariz aumentou 4,29% (*p* = 0,012).

**Conclusão:**

A curto prazo, a expansão rápida da maxila proporcionou um aumento estatisticamente significativo nas dimensões dos tecidos moles do nariz.

## Introduction and background

Maxillary atresia is considered a form of skeletal deformity characterized by a discrepancy in the maxilla/mandible relationship in the transverse plane which may lead to a posterior crossbite.^1^, ^2^ This clinical condition can cause many problems such as developmental abnormalities of the face and of occlusion, mouth breathing,^3^, ^4^ premature teeth loss and even postural problems involving irregular development of the body.^5^, ^6^, ^7^

Angell,^1^, ^2^ in 1860, was the first researcher to describe the possibility of opening the mid-palatal suture to achieve transverse maxillary correction, however, it was Hass^8^, ^9^ who published the first studies that clarified the real benefits of this treatment modalities. From Hass’ studies the utilized methods for rapid maxillary expansion became clearer and more standardized.^10^

Since then, innumerable experiments have been conducted demonstrating the importance of rapid maxillary expansion (RME) in facial development and occlusion.

In his previous studies, Haas pointed out the positive results/aspects in the nasal cavity after using the appliance. Later on, it was proved that, although there was a narrow anatomic relationship between the maxilla and the nasal cavity,^11^ RME was capable of changing this nasal physiology and anatomy.^7^, ^11^, ^12^ In many cases, it could improve breathing patterns by reducing the resistance of the nasal airway,^7^, ^13^ and thus substituting nasal breathing for a mouth breathing pattern in many patients.

For many years, the skeletal effects of RME were the main focus of the researchers but, some studies indicated that the soft tissues of the face, including the nose, followed the skeletal changes after the procedure,^13^, ^14^ causing possible effects on facial aesthetics^15^, ^16^ and thus interfering in the stability of the results achieved through skeletal expansion.^13^, ^14^

Berger et al.^15^ published the first reports on changes to nasal soft tissues, using digital photography to demonstrate a significant increase of 2 mm in the width of the nose after RME. This contrasted with results published by Johnson et al.^17^ who assessed the width of nasal soft tissues using high precision calipers and did not find significant differences between the periods, before and after RME. Karaman et al.^14^ conducted studies using lateral cephalometry, reporting that the length of the soft tissue of the nose tended to increase in line with the forward orthopedic displacement of the maxilla (Point A) during RME. Kiliç et al.^18^ also employed lateral cephalometry and reported similar results.

Kim et al.^19^ and Kulbersh et al.^13^ published the first study that evaluated changes of the soft tissues of the nose using a cone beam computed tomography (CBCT), which was considered the most precise diagnostic method for this type of research.^11^, ^13^ Both studies showed that the width of the soft tissues of the nose underwent a significant increase.

Magnusson et al.^16^ employed spiral computed tomography (CT) to assess the nasal soft tissues and documented that all dimensions increased with forward and downward displacement of soft tissues. Notwithstanding, they concluded that the largest changes were in the width of the nose. In one of the most recent studies conducted using CBCT, Yilmaz and Kucukkeles^20^ reported statistically significant changes in the width of the nose, but, in agreement with findings reported by Berger et al.,^15^ the increase in length proved to be without clinical or esthetic relevance.

It can be noticed that there are few studies in the scientific literature reporting the dimensional changes of the soft tissues of the nose after RME, increasing our motivation in the search for more research that can add useful and pertinent information to the theme.

The objectives of this study were to evaluate the dimensional changes of the nasal soft tissues after RME in all three planes (height, width and length) by using a multislice computed tomography (CT). And second, to determine whether the changes really took place, in what extension and if there is statistical significance to justify all the concern in orthodontics/orthopedics clinical practice with the effects of the RME procedure on nasal soft tissues.

## Materials and methods

This was a retrospective study of 30 patients divided in 2 groups: a Treatment Group (TG), underwent RME (20 patients, 10 of which were male and 10 female, with a *MA* of 8.9 years and a SD of 2.16, ranging from 6.5 to 12.5 years) and a Control Group (CG) (10 patients, 5 of which were male and 5 female, with a *MA* of 9.2 years, SD of 2.17, ranging from 6.11 to 13.7 years). All patients had maxillary atresia (sufficient level of maxillary atresia to indicate RME) and mouth breathing, diagnosed by otolaryngologists (mouth breathing) and orthodontists (maxillary atresia). The patients of the TG were treated with the aid of a Hyrax maxillary expander following the clinic's standard protocol of six activations in the early treatment and two daily activations, which was conducted until the upper bucal alveolar edge became transversely compatible to the WALA edge (Area of the greater transverse width, at the alveolar dental junction of the mandible). Computed tomography scans were taken at two different times: (T1) before RME and after 3 months wearing the device (T2). The patients of the CG underwent the same CT examinations (T1 and T2) at similar periods of time to those in the TG (3 months between them). It is important to clarify that all CT scans where acquired with proper prescription and pertinent authorization, and since this research was controlled with an already-existing database, no human beings were exposed to any quantity of ionizing ration radiation solely for the purposes of conducting this study. All patients were evaluated by a multi-disciplinary team and the diagnoses were made through a standardized questionnaire, as well as by a otolaryngological and orthodontic evaluation. Syndromic patients or patients with craniofacial abnormalities such as Pierre Robin and Treacher Collins, among others, and patients with dental or periodontal changes were excluded from the study. This study was approved by the Committee for Ethics in Institutional Research (registered under n° 164761), and by Clinical Trial (ID: CRB-ORTO3).

The width, height and length of nasal soft tissues were measured using anatomic landmarks defined in the global literature,^16^, ^20^, ^21^, ^22^ which are listed in Table 1 with their descriptions.

**Table 1:** Soft tissue landmarks.

Nasion (N′)	Point on the soft tissue midline that directly covers the hard tissue in the direction of the skeletal Nasion (N).
Pronasale (Prn)	The most prominent point of the nose located on the midline.
Alar (Al)	The most lateral point of the outside of each nostril.
Alar curvature (Ca)	Point of insertion into the soft tissue at each alar base.
Subnasale (Sn)	The midpoint between the junction of the lower margin of the nasal septum and the upper lip, on the midline

**Figure 1:**
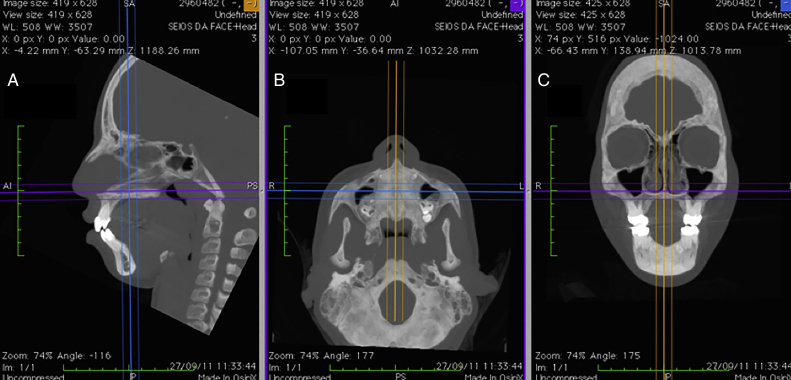
Multiplanar images (A) sagittal, (B) axial and (C) coronal, with contrast settings optimized for viewing the soft tissues of the nose.

Measurements before RME and after RME, in the TG, and measurements before T1 and T2, in the CG, were taken using OsiriX MD software (FDA approved, version 1.4.2; Pixmeo, Geneva, Switzerland), which offers the possibility of acquiring multiplanar slices (sagittal, axial and coronal) from the CT images. Using the program's dedicated tools, it is possible to define the optimum settings for contrast, select different density filters and apply transparency, resulting in perfect visualization of the soft tissues on sagittal, axial and coronal images (Fig. 1).

**Figure 2:**
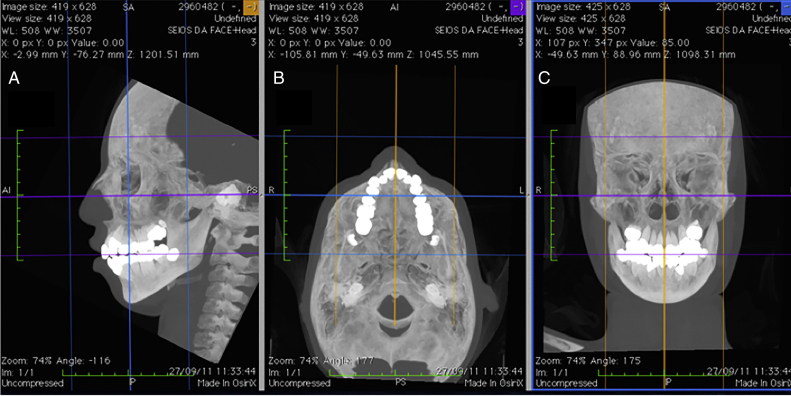
Repositioning the head using the program's horizontal and vertical reference lines in the (A) sagittal, (B) axial and (C) coronal planes.

In order to guarantee accurate measurements between the chosen landmarks, the patients’ heads were repositioned before taking the measurements, using the program's horizontal and vertical reference lines, following methodology described by Cevidanes et al.^23^ (Fig. 2).

**Figure 3:**
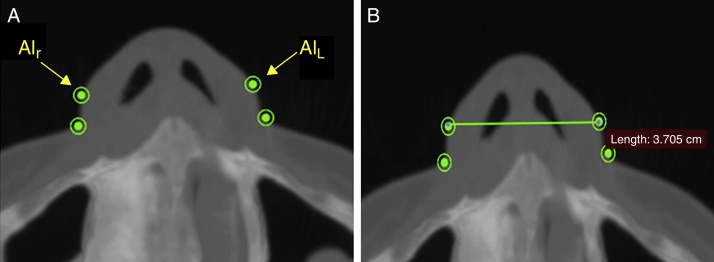
Measuring the alar width, in soft tissue on an axial image. Connecting points Al_r_ and Al_L_.

**Figure 4:**
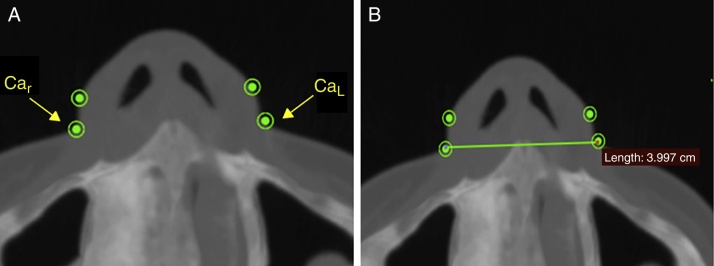
Measuring the alar base width at the soft tissue insertion on an axial image. Connecting points Ca_r_ and Ca_L_.

**Figure 5:**
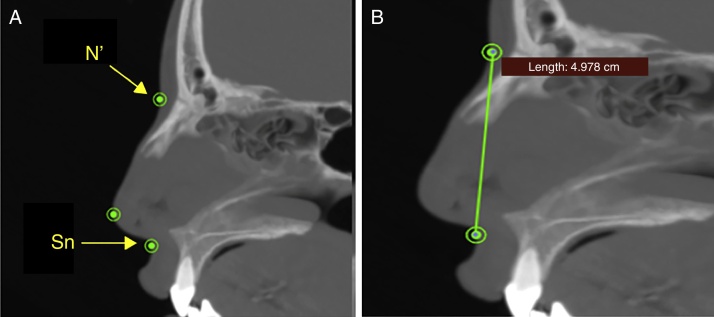
Measuring the height of the soft tissue of the nose on a sagittal image. Connecting points N′ and Sn.

**Figure 6:**
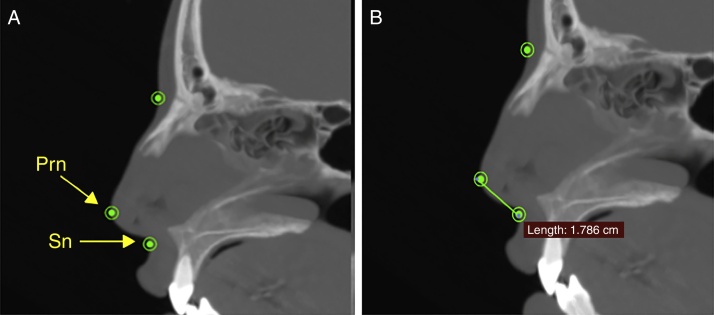
Measuring the length of the soft tissue of the nose on a sagittal image. Connecting points Prn and Sn.

The width of the soft tissue of the nose was measured on axial images at two different points, first by measuring the linear distance (in mm) between points Al_r_ and Al_L_ (alar width – Fig. 3) and second by measuring the distance between points Ca_r_ and Ca_L_ (alar base width – Fig. 4). Height was measured on sagittal images by taking the distance (in mm) between points N′ and Sn (Fig. 5), and length, also measured on sagittal images, was measured as the linear distance (in mm) from point Prn to point Sn (Fig. 6).

### Statistical analysis

Analysis and statistical treatment of data was accomplished using the Statistical Package for the Social Sciences (SPSS), version 22 for Windows.^24^ Measurement results are provided in millimeters (mm) and expressed as Means (M) and standard deviations in the form M ± SD.

To verify the suitability of the sample, the dimension of the effect (*d*) was calculated with a significance level of 5% (*α* = 0.05, being *α* the Type I error) and one power of the test (1 − *β*, being *β* the Type II error) of 80%. All calculations were made with the software G*Power^25^ and the classifications to the dimension effect proposed by Cohen (1992): *d* = 0.2 – small effect; *d* = 0.5 – médium effect; *d* = 0.8 – large effect, were given consideration. The sample (*n* = 20) secured the identification of the small/médium (*d* = 0.46), with a effect power of 80% and a significance level of 5%.

Normality of the data was verified using the Shapiro–Wilk test. Since normality was confirmed for all variables, parametric tests were used for statistical analyses: Student's *t* test for paired samples was used to test for significant differences between measurements taken before and after rapid maxillary expansion (RME).

Intra-examiner reliability was evaluated using Student's *t* test for paired samples and intraclass correlation coefficients (ICC). The results of statistical tests were judged against a significance level of 5%.

For the analysis of the comparison between T1 and T2 values in both groups, and for the comparison between the groups, the Student's *t*-test for paired samples was used.

## Results

The significance values (*p*) from the Shapiro–Wilk test used to analyze normality of data were greater than or equal to 0.05 for all variables. On this basis the null hypothesis of the test was not rejected to a significant level of 5% and it was therefore assumed that all data had normal distribution (Table 2).

**Table 2:** Results of the tests of normality of data: significance values (*p*) according to the Shapiro–Wilk test.

Variable	1st measurement		2nd measurement	
	Before RME (*p*)	After RME (*p*)	Before RME (*p*)	After RME (*p*)
Alar base width	0.886	0.378	0.684	0.850
Alar width	0.980	0.215	0.657	0.233
Height of soft tissue of the nose	0.115	0.097	0.245	0.093
Length of soft tissue of the nose	0.213	0.489	0.086	0.384

For the purposes of intra-examiner reliability assessment, the measurements of CT scans taken before and after RME were repeated by the same examiner 30 days after the first measurements were registered. All CT's were numbered without the observer knowledge if he was measuring the group pre or post RME.

After all measurements, every CT was organized properly in its corresponding group. The results of Student's *t* test for paired samples showed that there were no statistically significant differences (*p* > 0.05) between the means of the first and second measurements (repetition with a 30 day interval) for any of the variables tested, either for CT scans conducted before RME or for CT scans after RME. For all variables ICC values were greater than 0.95 (close to 1), indicating excellent consistency between results for the first and second measurements (Table 3). Taken together, these results guarantee excellent intra-examiner reliability for the measurements taken.

**Table 3:** Results of test of intra-examiner reliability: Student's *t* test for paired samples and intraclass correlation coefficients (measurements in mm).

Variable	1st measurement	2nd measurement	Difference	*p*^a^	ICC^b^
*Tomography before RME*					
Alar base width	33.11 ± 1.87	33.06 ± 1.85	−0.04	0.700	0.967
Alar width	33.25 ± 2.24	33.16 ± 2.41	−0.09	0.480	0.970
Height of soft tissue of the nose	49.03 ± 4.08	48.96 ± 3.88	−0.07	0.659	0.984
Length of soft tissue of the nose	15.88 ± 1.54	15.74 ± 1.49	−0.14	0.203	0.953

*Tomography after RME*					
Alar base width	33.93 ± 2.24	33.81 ± 2.08	−0.12	0.299	0.973
Alar width	33.77 ± 2.63	33.73 ± 2.70	−0.04	0.688	0.985
Height of soft tissue of the nose	49.82 ± 4.16	49.97 ± 4.19	0.16	0.497	0.970
Length of soft tissue of the nose	16.33 ± 1.94	16.32 ± 1.94	−0.01	0.940	0.950

Results expressed as mean ± standard deviation.

a *p*, significance according to Student's *t* test for paired samples.

b Intraclass correlation coefficients.

Analysis of the effects of RME in the TG and the analysis of the effects between T1 and T2 in the CG, as well as the analysis of the effects between the two groups, were taken using the first set of measurements from scans, and the results of these analysis are listed in Table 4.

**Table 4:** Comparison between measurements before RME and after RME and between Treatment Group (TG) and Control Group (CG) (measurements in mm).

Variable	Group	Before RME	After RME	Difference before after		*p*^a^ (before RME − after RME)
				Mean	%	
Alar base width	TG	33.36 ± 1.95	34.99 ± 1.90	1.62	+4.87%	0.004
	CG	32.85 ± 1.86	32.87 ± 2.12	0.03	−0.08%	0.938
*p*^b^ (between groups)		0.550	0.030			

Alar width	TG	33.65 ± 2.54	35.01 ± 2.29	1.36	+4.04%	0.004
	CG	32.85 ± 1.95	32.52 ± 2.42	−0.33	−0.99%	0.362
*p*^b^ (between groups)		0.438	0.030			

Height of soft tissue of the nose	TG	48.51 ± 4.22	50.98 ± 4.32	2.47	+4.84%	0.003
	CG	46.77 ± 4.06	46.92 ± 3.94	0.15	+0.32%	0.228
*p*^b^ (between groups)		0.509	0.028			

Length of soft tissue of the nose	TG	16.52 ± 1.53	17.23 ± 1.84	0.71	+4.29%	0.001
	CG	15.23 ± 1.33	15.43 ± 1.66	0.19	+1.27%	0.491
*p*^b^ (between groups)		0.061	0.034			

Results expressed as mean ± standard deviation.

a *p*, significance according to Student's *t* test for paired samples (differences between before and after).

b *p*, significance according to Student's *t* test for independent samples (differences between TG and CG).

A global evaluation of the results showed that there was, in the TG, a statistically significant increase in all four measurements from before RME to after RME (*p* < 0.05), whereas in the CG no significant changes was observed between T1 and T2 times (*p* > 0.05). Furthermore, comparison between both groups showed a statistically significant difference (*p* < 0.05), revealing that RME induced increases in the values of alar base width, alar width, height of soft tissue of the nose and length of soft tissue of the nose.

The mean value for alar base width, in the TG, increased significantly (*p* = 0.004) from 33.36 ± 1.95 mm before RME to 34.99 ± 1.90 mm after RME, which is equivalent to a mean increase of 4.87%. In the CG, no significant difference was observed (*p* = 0.938) between means of T1 (32.85 ± 1.86) and T2 (32.87 ± 2.12).

The mean Alar width measurement, in the TG, increased 4.04%, from 33.65 ± 2.54 mm to 35.01 ± 2.29 mm, which was a statistically significant increase (*p* = 0.004). In the CG, no significant difference was observed (*p* = 0.362) between means of T1 (32.85 ± 1.95) and T2 (32.52 ± 2.42).

The height of the soft tissue of the nose, in the TG, increased significantly (*p* = 0.003), from 48.51 ± 4.22 mm before RME to 50.98 ± 4.32 mm after RME, which is the equivalent of 4.84%. In the CG, no significant difference was observed (*p* = 0.228) between means of T1 (46.77 ± 4.06) and T2 (46.92 ± 3.94).

The mean length of the soft tissue of the nose, in the TG, increased by 4.29%, from 16.52 ± 1.53 mm to 17.23 ± 1.84 mm, which is a statistically significant increase (*p* = 0.001). In the CG, no significant difference was observed (*p* = 0.491) between means of T1 (15.23 ± 1.33) and T2 (15.43 ± 1.66).

## Discussion

Since the first reports were published by Angell^1^, ^2^ and Haas,^8^, ^9^, ^10^ numerous studies have clearly demonstrated that RME is capable of altering the physiology and anatomy of the nasal cavity.^7^, ^11^, ^12^, ^13^

The soft tissues of the face, including the nose, have been recently investigated because of the esthetic consequences and also in relation to the stability of the results achieved using RME.^13^, ^14^, ^15^, ^16^

The first studies, focused on changes to nasal soft tissues, were conducted, using measurements on digital photographs, before and after RME,^15^ directly on patients’ faces using high-precision calipers^17^ or on digital cephalometry.^14^, ^18^ These studies analyzed only the changes to width^15^, ^17^ and length.^14^, ^18^ With regard to soft tissue width, Berger et al.^15^ found a mean increase of 2 mm after RME. Our study demonstrated similar results with mean increases of 1.62 mm in alar base width and 1.36 mm in alar width. Both of these results were statistically significant, in contrast with results reported by Johnson et al.,^17^ who also identified increases in soft tissue width, but, according to their results, without statistical significance. With regard to the length of the soft tissue of the nose, our study demonstrated a significant mean increase of 4.29% among patients after RME, which is in agreement with outcomes published by Karaman et al.^14^ and Kiliç et al.^18^

Studies undertaken using cone beam computed tomography (CBCT),^13^, ^19^, ^20^ showed that RME resulted in significant increases in the transversal dimensions of the soft tissues of the nose, which agrees with our results, but, in contrast with our findings, they found that increases in length were not statistically relevant. This discrepancy could occurred because the CBCT has lower radiation dose and is not recommended for soft tissue measurement. However, Magnussen et al.^16^ used spiral computed tomography (CT) scans to measure nasal soft tissues, in common with our study. These authors concluded that although there were changes to all of the dimensions of the nose, only differences in width measurements were significant, which does not agree with our results, since we demonstrated statistically significant differences in all variables studied. We believe that these differences in the results occurred because Magnussen et al. carried out their study with patients who underwent surgically assisted RME, with patients outside of the facial skull growth phase, while our study was performed only with orthopedic RME in patients which were in the active phase of growth.

Practically none of the studies cited assessed the height of the soft tissue of the nose. The majority only studied transverse changes and few measured length. In our study, we also investigated the possibility of changes to the height of the soft tissue of the nose, finding that there had been a significant increase, of approximately 4.84%, after RME.

Even when studying patients in the growth phase, we believe that the changes observed in our study, occurred solely due to the action of RME, since the time of evaluation between T1 and T2 times was only 3 months, would be insufficient for a significant interference of the growth in the obtained results.

We should make clear that all patients that took part of this research underwent the CT exams in the same place, with the same equipment and with the same operator, respecting the ALARA principle^26^, ^27^ (As Low As Reasonably Achievable) to each patient.

It is also important to clarify that, after the end of the study, the patients of the CG were properly treated with the same procedures of the TG, without any prejudice to them, due to the small time of 3 months between T1 and T2 times.

Our study utilized an already existing database with the pertinent authorizations and approved by the ethics committee.

## Conclusions

Mouth breathing children after rapid maxillary expansion showed a short-term statistically significant increase in measurements of alar base width, at the point of soft tissue insertion, alar width, height of soft tissue of the nose and length of soft tissue of the nose.

## Conflicts of interest

The authors declare no conflicts of interest.
